# A Comparative Study between Lycorine and Galantamine Abilities to Interact with AMYLOID β and Reduce In Vitro Neurotoxicity

**DOI:** 10.3390/ijms24032500

**Published:** 2023-01-28

**Authors:** Arian Kola, Stefania Lamponi, Francesco Currò, Daniela Valensin

**Affiliations:** 1 Department of Biotechnology, Chemistry and Pharmacy, University of Siena, Via Aldo Moro 2, 53100 Siena, Italy; 2 CIRMMP, Via Luigi Sacconi 6, 50019 Firenze, Italy

**Keywords:** amyloid-β, alkaloid, lycorine, galantamine, *Amaryllidaceae*, ligand-protein interaction, SH-SY5Y cell line

## Abstract

Galantamine is a natural alkaloid extracted from the *Amaryllidaceae* plants and is used as the active ingredient of a drug approved for the treatment of the early stages of Alzheimer’s disease. It mainly acts as an acetylcholinesterase (AChE) inhibitor, increasing concentrations of the acetylcholine neurotransmitter. Recent cellular studies have also shown the ability of galantamine to protect SH-SY5Y cell lines against amyloid-β (Aβ)-induced toxicity. Such investigations have supported and validated further in-depth studies for understanding the chemical and molecular features associated with galantamine-protective abilities. In addition to galantamine, other natural alkaloids are known to possess AChE inhibitory activity; among them lycorine has been extensively investigated for its antibacterial, anti-inflammatory and antitumoral activities as well. Despite its interesting biological properties, lycorine’s neuroprotective functions against Aβ-induced damages have not been explored so far. In this research study, the ability of galantamine and lycorine to suppress Aβ-induced in vitro neuronal toxicity was evaluated by investigating the chemical interactions of the two alkaloids with Aβ peptide. A multi-technique spectroscopic analysis and cellular cytotoxicity assays were applied to obtain new insights on these molecular associations. The comparison between the behaviors exhibited by the two alkaloids indicates that both compounds possess analogue abilities to interact with the amyloidogenic peptide and protect cells.

## 1. Introduction

Alzheimer’s Disease (AD) consists of pathological neurostructural degeneration proceeding in a slow and progressive way until the death of the patient [1]. Despite this pathology being discovered at the beginning of the twentieth century, no cure is available so far and its growing incidence worldwide is worrisome [2]. This phenomenon is correlated to the increased life expectancy which leads to a major exposure to oxidative factors altering the regular balance of neuronal structures. 

Currently more than 55 million people worldwide are affected by dementia, with AD being the most prevalent form, and there are nearly 10 million new cases every year [2]. Moreover, AD patients require health care characterized by high costs for both families and national health care systems [3].

The causes of AD have not yet been identified certainly, but nowadays the amyloidogenic pathway hypothesis is the most accredited one [4,5]. The incorrect proteolytic cutting of the amyloid precursor protein (APP) by specific secretases leads to the formation of insoluble amyloid β peptide (Aβ), 40 and/or 42 amino acids long, which is able to give rise to deposits of extracellular Aβ fibrils [6,7]. Fibril deposition is preceded by the formation of Aβ soluble oligomers and insoluble aggregates which are very toxic to neuronal cells along with the elevated level of reactive oxygen species (ROS) and metal dyshomeostasis (Cu, Fe, Zn) well known as typical AD features [8,9,10,11,12,13,14,15,16]. 

To date there is no effective cure for AD with the available treatments capable only of alleviating the initial pathological symptoms. These treatments include β and γ secretase inhibitors, which are able to interfere with the formation of the amyloidogenic Aβ peptide, anti-inflammatory drugs and antioxidants capable of radical scavenging [4,17,18]. Recently, four anti-Aβ antibodies targeting and reducing amyloid deposits have been discovered [19]; among these the FDA, but not the EMA, has recently approved Aducanumab for the treatment of AD mild cognitive impairment (MCI) or mild dementia [20]. Besides that, the scientific community have put a lot of efforts to identify new molecules able to inhibit the aggregation of Aβ and to understand the role played by pathogenic protein conformational changes in neurodegeneration [21,22].

In the early AD phase, the most used drugs are generally acetylcholinesterase (AChE) inhibitors which promote an increase of acetylcholine levels in the synaptic junction, allowing a better cholinergic neurotransmission. Within this group of compounds are Tacrine, Donepezil, Rivastigmine and Galantamine. Tacrine was synthesized by Adrien Albert at the University of Sydney in 1949. It was the first AChE inhibitor approved by the FDA (1993), but its use is limited nowadays because of its low bioavailability and tolerance [23]. Donepezil is a synthetic compound developed by Eisai. It was approved for medical use by the FDA in 1996 [24] and is widely used in the USA where it was the 120th most prescribed medication in 2019. Rivastigmine, approved by the FDA in 2000, is a semi-synthetic derivative of the alkaloid physostigmine developed by Marta Weinstock-Rosin of the Department of Pharmacology at the Hebrew University of Jerusalem [25]. Lastly, the alkaloid galantamine (GAL) was approved by the FDA in 2001 and it is the only molecule among the above-mentioned AChE inhibitors, which is naturally found [26]. Natural compounds, present as bioactive constituents in traditional medicinal plants, have been used for a long time, and nowadays they are being introduced as therapeutic agents in several diseases. Among them, natural neuroprotective alkaloids, polyphenols, polysaccharides and terpenoids have been deeply investigated for the treatment of AD [27,28,29,30]. 

GAL is derived from the extract of *Amaryllidaceae* plants which produce a series of unique isoquinoline alkaloids with relevant pharmacological properties [31,32,33,34,35,36]. So far, the exact mechanism by which GAL exerts its pharmacological activity is not fully understood: GAL has been proposed as a reversible, competitive and selective AChE inhibitor, and as a ligand of the nicotinic acetylcholine receptors leading to an increase in neurotransmitters secretion [37].

Besides the cholinergic modulation exhibited by GAL, further studies have revealed the ability of this alkaloid to fight off Aβ-induced neuronal toxicity in SH-SY5Y cell lines by acting as an antioxidant, radical scavenging, neuroprotective, anti-aggregation and antigenotoxic compound [38,39,40,41,42,43,44].

In addition to GAL, other *Amaryllidaceae* alkaloids have strong AChE inhibitory activity. Among these haemanthamine, narciclasine, crinine and lycorine have recently been considered [32,45,46,47]. All of these alkaloids, strictly related to the leading compound galantamine, have interesting features and deserve to be further investigated for promoting new natural molecules for AD treatment. 

Lycorine (LYC) is a very interesting molecule since it exhibits a broad spectrum of biological functions [48], including antibacterial [49], anti-inflammatory [50,51] and antitumoral [52,53,54] and, together with GAL, it has the greatest AChE inhibitory activity [46,47,55,56]. Furthermore, a recent study conducted by Zhang et al. corroborates LYC’s ability to suppress stress-induced premature cellular senescence by stabilizing the genome of human cells, potentially delaying the onset of age-associated diseases such as AD [57]. Castillo et al. published a study performed on SH-SY5Y cells poisoned with Aβ peptide, where the co-administration of GAL and LYC even reduced the genotoxicity and cell death compared to the single administration [58]. 

In this study a comparative analysis was carried out by evaluating the neuroprotective, antioxidant and Aβ-binding properties of these two *Amaryllidaceae* alkaloids. A combined approach consisting of NMR, CD and UV-Vis spectroscopy was applied to describe GAL and LYC molecular activities towards the pathogenic Aβ peptide. On the other hand, cytotoxicity cellular studies were performed using differentiated human SH-SY5Y neuroblastoma cells exposed to toxic Aβ concentrations. 

## 2. Results

### 2.1. Atomic and Molecular Interactions between GAL/LYC and Aβ 

The interaction between the two alkaloid molecules (GAL and LYC) and Aβ40 was investigated by applying both ligand- and peptide-based NMR approaches [59]. In the former case the variations of the NMR parameters of GAL and LYC protons in presence of substoichiometric Aβ40 amounts were determined. On the other hand, an almost equimolar Aβ40:alkaloid ratio was used to map peptide regions involved in the binding sites. For the NMR analysis, the Aβ40 isoform is usually preferred to Aβ42 [60,61], the latter being more prone to aggregate and thus is less appropriate for NMR measurements.

**Figure 1:**
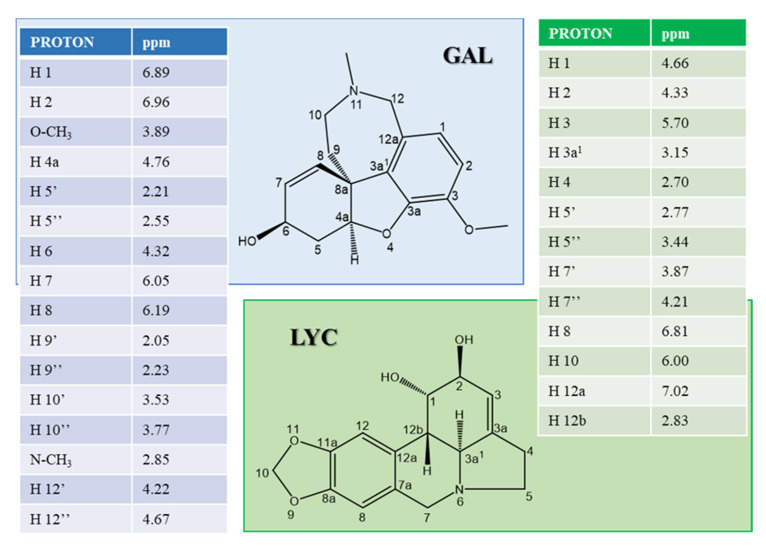
^1^H chemical shift values (ppm) of GAL (**left**) and LYC (**right**) protons. NMR 1D spectra were recorded on a solution of alkaloid 0.25 mM in 20 mM phosphate buffer, pH 7.5, T = 298 K.

The chemical shift assignment of GAL and LYC at pH 7.5 and room temperature was obtained by combining 1D and 2D NMR spectra and the corresponding data are reported in Figure 1.

**Figure 2:**
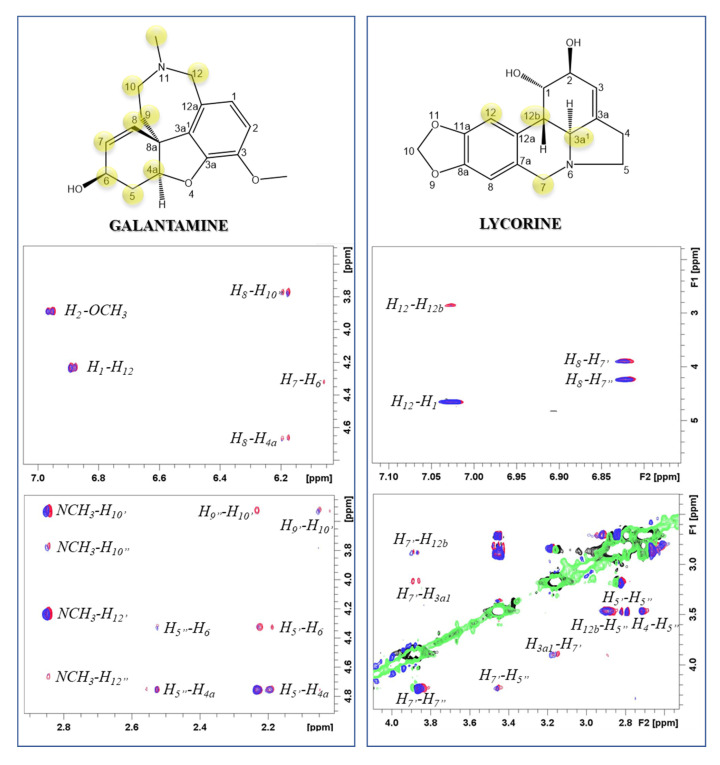
Selected regions of ^1^H-^1^H NOESY spectra of GAL (**left**) and LYC (**right**) in absence (red contours) and in presence of 0.3 equivalent (eqs.) of Aβ40 (blue contours). Alkaloid concentration 0.25 mM, Aβ40 concentration 0.083 mM, phosphate buffer 20 mM, pH 7.5, T = 298 K.

^1^H-^1^H NOESY NMR spectra of both alkaloids are characterized by NOE correlations with the opposite sign to the diagonal signals in agreement with the expected molecular tumbling of the analyzed compounds (positive NOEs). Transferred NOE (tr-NOE) experiments are commonly employed to characterize ligand-protein binding [62]. According to this method, tr-NOEs measured upon addition of Aβ40 to each alkaloid solution gave evidence of interaction for both GAL and LYC (Figure 2). In fact, the cross-peak’s intensities of both GAL and LYC protons are selectively decreased upon Aβ40 addition, indicating that the alkaloid motion in solution is slowed down by the association with the peptide [63,64]. The most affected GAL proton signals belong to the tetrahydroazepine (H9, H10, H12 and NCH3) and ciclohexenol (H8, H7, H6, H5, H4a and H8a) moieties (Figure 2). Similarly, the dihydroisoquinoline region contains the most perturbed LYC protons (H3a1, H7’, H12, H12b) (Figure 2). The detected NOE variations, together with the absence of significative chemical shift variations, indicate that both alkaloids form weak complexes with Aβ [63]. In agreement with our data, previous NMR difference spectroscopy analysis on GAL-Aβ40 interactions identified tetrahydroazepine and ciclohexenol portions as the sites interacting with Aβ40 [42].

NMR spectra of GAL and LYC were also performed by incubating Aβ40-alkaloid solutions at room temperature at different time intervals to evaluate the impact of peptide aggregation on alkaloid interactions. It is in fact well known that both Aβ40 and Aβ42 aqueous solutions undergo fast aggregation phenomena at physiological pH. Our findings indicate that both Aβ40-alkaloid systems are almost unchanged after 24 h; on the other hand, slight chemical shift variations are observed at 48 h, especially for LYC, exhibiting the largest effects on H3a1 and H7’, in the area close to the nitrogen atom at position 6 (Supplementary Figure S1). Finally, tr-NOE spectra collected on Aβ40-alkaloid 48 h incubated samples confirmed the results observed on the freshly prepared solutions indicating that both GAL and LYC are able to interact with different Aβ40 forms.

**Figure 3:**
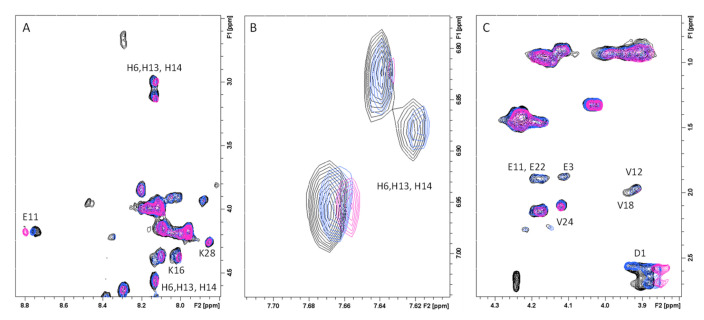
Selected regions, (**A)** fingerprint region; (**B**) aromatic region; (**C**) aliphatic region, of ^1^H-^1^H TOCSY spectra of Aβ40 in absence (magenta contours) and in presence of 0.8 eqs. of GAL (blue contours) and LYC (black contours). Aβ40 concentration 0.18 mM, EtOD(60)/H_2_O(40) (phosphate buffer 20 mM) mixture, T = 298 K.

**Figure 4:**
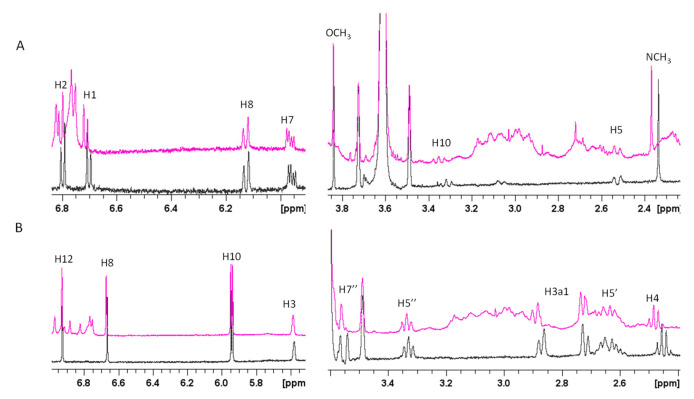
Selected regions of ^1^H spectra of (**A**). GAL and (**B**). LYC in absence (black lines) and in presence of 1.2 eqs. of Aβ40 (magenta lines). Alkaloid concentration 0.15 mM, Aβ40 concentration 0.18 mM EtOD(60)/H_2_O(40) (phosphate buffer 20 mM) mixture, T = 298 K.

In order to investigate the Aβ regions involved in GAL/LYC interactions 1D and 2D NMR spectra of Aβ40 were performed as well (peptide-based approach). Because of the peptide aggregation propensity at relatively high NMR concentrations (180 μM), Aβ40 was dissolved in EtOD/H_2_O (60/40) mixture, well known to slow down Aβ aggregation [65]. The comparison of 1D ^1^H and 2D ^1^H-^1^H TOCSY experiments of Aβ40, in the absence and in the presence of almost equimolar GAL/LYC concentrations (150 μM), showed the ability of both alkaloids to induce selective changes on Aβ40 resonances (Figure 3). The most affected signals belong to the three histidine (His6, His13 and His14), the N-terminal Asp1 and Glu11 NH, whose signals are characterized by slight chemical shift variations and line narrowing. Lys16 and Lys28 correlations also exhibited minor changes upon alkaloids addition (Figure 3). All these data strongly indicate the N-terminal part as the Aβ region involved in GAL/LYC interactions. Furthermore, the analysis of 1D spectra clearly indicated chemical shift variations on GAL/LYC protons in the free and Aβ40 bound forms (Figure 4). For GAL, the most affected signals belong to NCH_3_, H1, H2, H7 and H10 with the largest effect on N-methyl protons (0.03 ppm). For LYC, H4, H7’ and H3a1 are the most shifted resonances in agreement with the data previously shown in Figure S1. On the other hand, the absence of shifts previously measured for GAL protons might be explained by considering the different GAL:Aβ40 ratio used for ligand and peptide-based experiments. 

### 2.2. GAL and LYC Effects on the Secondary Structure of Amyloid-β

**Figure 5:**
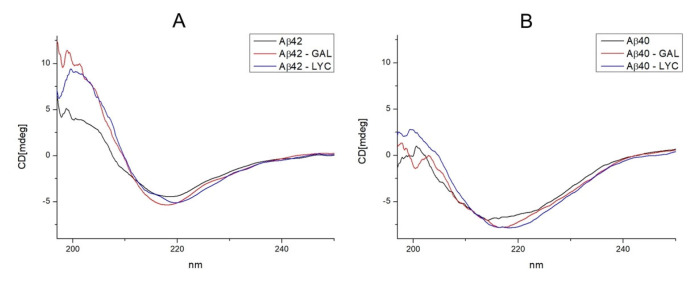
CD spectra of Aβ42 (**A**) and Aβ40 (**B**) in absence (black lines) and in presence of 0.8 GAL (red lines) and LYC (blue lines) eqs. Aβ42/Aβ40 concentration 3 µM, GAL concentration 2.4 µM, LYC concentration 2.4 µM, phosphate buffer 1 mM, T = 298 K.

Circular dichroism is a well applied technique to investigate the changes of the secondary structures content in amyloidogenic proteins [66,67,68]. The effect of metal ions, small ligands and natural compounds on Aβ structure can easily be evaluated by comparing far-UV CD spectra before and after the addition of the investigated compound. According to that, CD spectra of Aβ42, Aβ42-GAL, Aβ42-LYC systems were measured to evaluate the alkaloid’s abilities to induce structural rearrangements in Aβ. Our findings indicate that both GAL and LYC addition leads to changes in CD absorption bands, as shown in Figure 5. Free Aβ42 peptide exhibited CD spectra with minimum absorption at 218 nm typical of β-sheet structure, alkaloid’s addition leads to a tiny increase in band intensity and a small shift to 221 nm for LYC only. Furthermore, analogues studies with Aβ40 peptide displayed a similar behavior, strongly indicating that both alkaloids are able to induce structural changes on both Aβ42 and Aβ40 isoforms (Figure 5).

### 2.3. Antioxidant abilities of GAL and LYC

**Figure 6:**
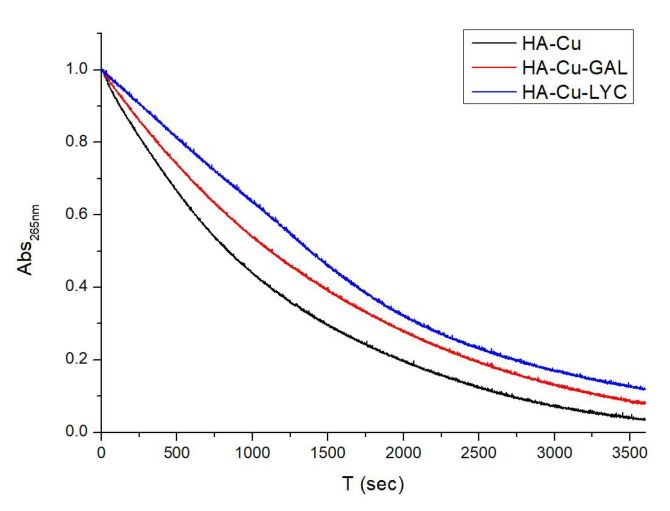
UV-Vis kinetic curve of ascorbate oxidation by Cu(II) ions in absence (black lines) and in presence of equimolar GAL (red lines) and LYC (blue lines) concentrations. Ascorbate concentration 50 µM, 200:1 ascorbate:copper(II) ratio, phosphate buffer 3 mM solution, pH 7.5, T = 298 K.

Previous studies on GAL pointed out its ability to reduce ROS release and to protect cells by lowering oxidative injury [38,39]. It is well known that copper and iron binding to Amyloid β (Aβ) leads to ROS production [9,69]. In vitro studies have supported the Cu-Aβ ability to catalyze the formation of H_2_O_2_ and HO^•^ species in the presence of O_2_ and a reducing agent, such as ascorbate [70]. In order to evaluate the antioxidant role played by GAL and LYC in copper/ascorbate systems, the ascorbate oxidation in presence of copper(II) was measured with and without both alkaloids. The ascorbate consumption at 265 nm was therefore monitored by UV-Vis spectroscopy, as previously reported [71]. The obtained kinetic curves shown in Figure 6 indicate that both GAL and LYC are able to delay the ascorbate oxidation by preventing ROS production, which usually occurs in copper/ascorbate systems. Notably the copper binding to both alkaloids was additionally evaluated in this study by means of NMR spectroscopy. Upon Cu(II) addition to GAL and LYC solutions no line broadening or chemical shift was observed, thus excluding Cu(II) interactions with both alkaloids.

### 2.4. The Protective Role of GAL and LYC against Aβ-Induced Cytotoxicity

Considering that differentiated SH-SY5Y cells are more susceptible to Aβ toxicity [72], two different Aβ42 concentrations (2 and 5 μM) were first tested in order to establish a model of Aβ-induced toxicity. As expected, both Aβ42 concentrations were found to be cytotoxic towards neuronal cells but to different degrees (Supplementary Figure S2). Of the two, the 5 µM concentration was found to drastically reduce the percentage of viable cells that was significantly different from the negative control (complete medium). On the other hand, the 2 μM concentration reduced the percentage of viable neuronal cells by about 46% compared to the negative control. For this reason, for further experiments with LYC and GAL the 2 µM concentration of Aβ42 was selected.

Differentiated SH-SY5Y cells were also incubated with different concentrations of LYC and GAL. Cell viability was analyzed after 24 h of contact with the test samples showing higher percentage of viable cells by decreasing both GAL and LYC concentrations (Supplementary Figure S3). The lowest GAL and LYC concentration showing the same percentage of viable cells as the negative control are 50 μM and 1.6 µM, respectively. These concentration values are therefore lacking cytotoxic effect against differentiated SH-SY5Y cells. 

**Figure 7:**
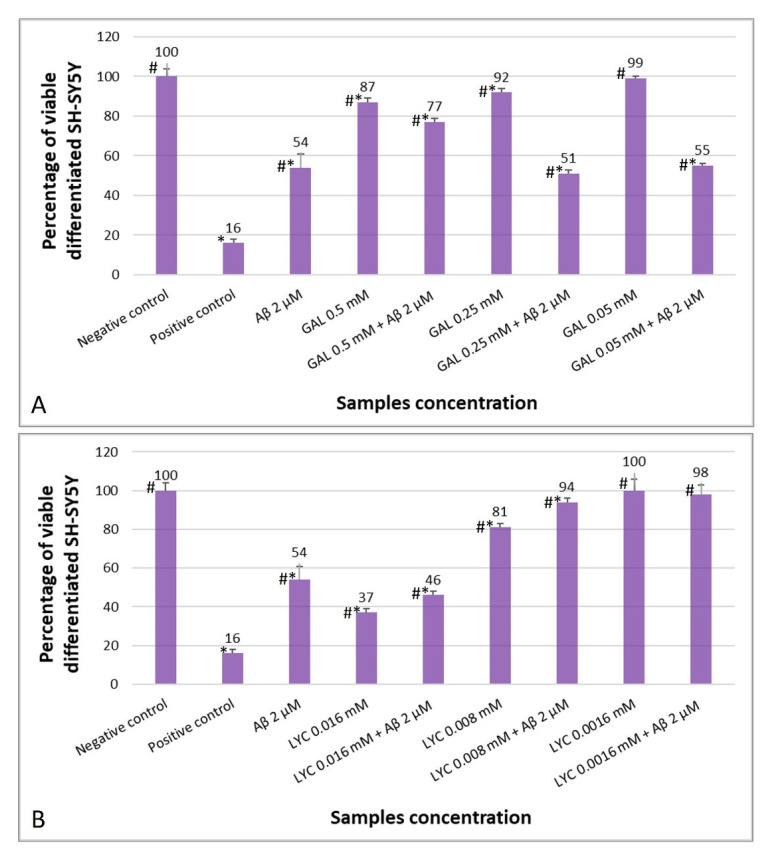
Effect of Aβ 2 µM towards viability of differentiated SH-SY5Y as a function of (**A**) GAL and (**B**) LYC concentration, as determined by the Neutral Red Uptake. Data are mean ± SD of three replicates for each sample. * Values are statistically different versus negative control (complete medium), *p* < 0.05. # Values are statistically different versus positive control (PVC-org.Sn), *p* < 0.05.

The effects of the two alkaloids on cells exposed to Aβ (2 μM) were further evaluated at different GAL and LYC concentrations, as shown in Figure 7A,B, respectively. Exposure of differentiated SH-SY5Y cells to GAL resulted in increased cell survival only at the highest estimated GAL concentration, i.e., 500 µM (77%) compared to Aβ alone (54%). By decreasing the concentration of GAL to 250 μM, cell viability was dramatically reduced to 51%, contrary to what was previously observed for GAL 250 µM alone, showing 92% cell viability. The same trend was observed with the lowest GAL concentration, i.e., 50 μM. In this case, cell survival in the presence of GAL alone was 99% while it was reduced to 55% when Aβ (2 µM) was added. So, GAL reduced the toxic effect of amyloid towards differentiated SH-SY5Y only at the highest concentration tested even though the GAL 500 μM-Aβ 2 µM resulted more toxic (77%) than the GAL alone (87%).

Treatment of differentiated SH-SY5Y cells with both LYC 16 μM and Aβ 2 µM reduced their viability to approximately 46% (Figure 7B). This value was not statistically different from viable cells in contact with Aβ 2 μM alone, but different from 16 µM LYC which reduced cell viability to 37%. By decreasing the LYC concentration to 8 μM, cell survival increased with both the alkaloid alone and with Aβ 2 μM (81 and 94%, respectively), but in presence of Aβ the cell survival rate was significantly higher (Figure 7B). Finally, at the lowest concentration tested (1.6 µM), LYC proved to be void of cytotoxic effects both without and with Aβ 2 μM (100 and 98%, respectively) (Figure 7B).

## 3. Discussion

In this study, the ability of GAL and LYC to interact with the amyloid β peptide was evaluated in order to contribute, from an atomic and molecular perspective, to the comprehension of the protective role exhibited by LYC and GAL. 

GAL ability to bind Aβ and inhibit its aggregation in vitro have been previously investigated by means of NMR difference spectroscopy indicating the occurrence of an early interaction between GAL and soluble Aβ [42]. The use of NMR spectroscopy to investigate molecular interactions is well accepted since this technique can give useful information on both strong and weak associations in solutions. In our research, NMR studies were performed by using the Aβ40 peptide which is less prone to aggregation than Aβ42 [73]. Upon substochiometric Aβ40 additions to GAL and LYC solutions, the NMR parameters of both alkaloids were measured at different incubation times (t = 0, 24 h and 48 h) to evaluate the ability of GAL and LYC to interact with different Aβ40 forms. According to previous studies monomeric and indistinguishable Aβ40/Aβ42 conformations are expected at t = 0, while at higher incubation times, the formation of aggregated amyloidogenic forms can be anticipated [73].

Our data indicate that both alkaloids weakly interact with the monomeric peptide as proved by the analysis of tr-NOEs (Figure 2); on the other hand, the chemical shift variations observed at longer incubation times (Figure S1) point out LYC ability to associate tighter with Aβ40 than GAL. This interaction might strongly interfere with the processes leading to Aβ40 aggregation thus explaining the behavior of LYC to protect SH-SY5Y cells from Aβ toxicity more effectively than GAL (Figure 7). 

Our findings indicate that the alkaloid regions exhibiting the largest effects upon Aβ40 addition are the tetrahydroazepine and the dihydroisoquinoline portions for GAL and LYC, respectively, highlighting that the Aβ40-alkaloid association is mediated by the tertiary amino group, positively charged at the applied experimental conditions (pKa GAL = 8.2 [74], pKa LYC = 7.8 [75]). The key role played by the azepine nitrogen atom was also pointed out by previous molecular dynamics studies on GAL-Aβ associations which support the formation of (i) a salt bridge between the positively charged N atom of GAL and the carboxylate group of Asp23 [76] and (ii) a peptide dimer formation stabilized by the N atom [77]. Both investigations agree with the occurrence of Aβ structural rearrangements upon GAL binding, thus explaining the alkaloid ability to interfere with the processes leading to peptide aggregation. The conformational Aβ changes upon alkaloids binding are well supported by our CD measurements (Figure 5), indicating that both Aβ40 and Aβ42 isoforms experience different secondary structure elements in the absence or presence of GAL/LYC.

Unlike GAL, so far, no data are available in the literature for LYC, being our study, the first investigation focused on the comprehension of the molecular mechanisms of LYC–Aβ interaction. However, considering that LYC and GAL share several similar structures such as (i) the tertiary amino group, (ii) the presence of hydroxyl groups, (iii) an aromatic ring and a double bond, (iv) a predominantly planar geometry and (v) almost identical molecular weight (GAL = 287.34 g/mol, LYC = 287.31 g/mol), a similar interaction mode is expected for LYC/GAL-Aβ association. On this regard, our NMR findings support that both alkaloids interact with the N-terminal region of Aβ40, where Asp1, His6, Glu11, His13 and His14 are the residues most perturbed by the presence of both alkaloids. This finding in addition to identifying the Aβ40 region responsible of GAL/LYC binding allows us to speculate that (i) monomeric Aβ42, differing from Aβ40 only in the last two amino-acids (Ile and Ala), holds an interaction mode similar to the shortest Aβ isoform and (ii) not only Asp23, but rather the negatively charged residues at the N-terminus (Asp1, Glu3, Asp7 and Glu11) take part in the GAL/LYC association. 

Finally, a fine comparison between the two alkaloid-Aβ interactions highlights the occurrence of a diverse GAL and LYC affinity for Aβ, as supported by the slightly more pronounced chemical shift variations exhibited by LYC (Figure S1 and Figure 4). This behavior might be justified by considering that LYC has a higher number of hydrogen bond donors and acceptors than GAL, thus providing a more efficient network to stabilize the alkaloid-peptide association. In fact, as expected, the LYC protons experiencing the largest chemical shift variations include those close to the hydroxyl groups (H12b and H3), while smaller effects are detected for the same protons in GAL (H5 and H7).

Interestingly, our data point out an improved LYC performance in reducing ascorbate oxidation, supporting its ability to more effectively prevent ROS production than GAL (Figure 6). Similarly, our studies showed that LYC is more effective than GAL in attenuating Aβ-induced cytotoxicity in differentiated human neuroblastoma SH-SY5Y cells, routinely chosen as in vitro AD model for neuron differentiation and neurodegenerative disorder research [78]. In the undifferentiated form, SH-SY5Y cells possess a neuroblast-like morphology, with non-polarized cell bodies and few truncated processes. On the other hand, treatments with specific differentiation-inducing agents lead to cellular morphologies more similar to primary neurons with long neuronal processes [79]. According to the used treatments, different cellular phenotypes are obtained. Retinoic acid, for example, induces SH-SY5Y differentiation primarily to a cholinergic neuron phenotype. Undifferentiated and differentiated SH-SY5Y cells can express both muscarinic and nicotinic acetylcholine receptors. However, retinoic acid treatment increases the number of muscarinic binding sites and leads to significantly higher acetylcholinesterase and choline acetyltransferase activities [80]. 

**Figure 8:**
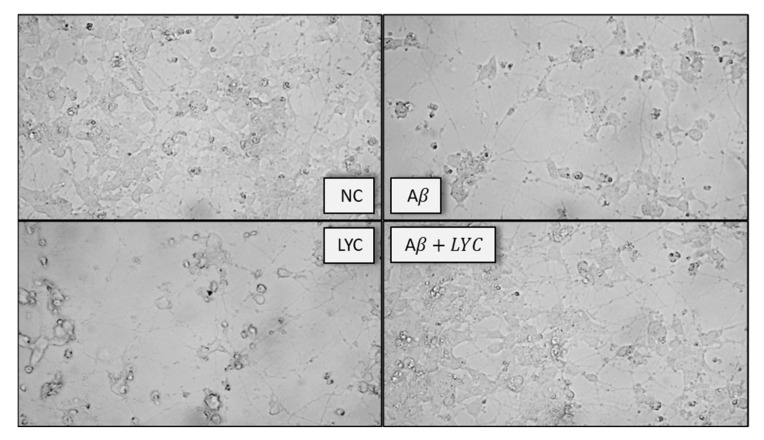
Optical microscope images of differentiate SH-SY5Y in contact with Negative Control (NC), Aβ42 2 µM; LYC 8 µM; Aβ42 2 µM + LYC 8 µM. Magnification 40x; scalebar 5 µm.

In our study, SH-SY5Y differentiation was accomplished by using the procedure reported by Shipley et al. developed to appropriately differentiate cells into neurons and to obtain the best possible representation of in vivo neuronal model [81]. Our results indicated that the vitality and morphology of SH-SY5Y cells are strongly altered upon Aβ42 exposure (Figure 8). Aβ42 is capable of wiping out most of the neuronal processes visible in healthy cells (negative control). In a similar way, both the cytotoxic and non-cytotoxic alkaloid concentrations reduce the finger-like projections connecting SH-SY5Y cells, as reported in Figure 8, showing the cellular morphology upon LYC (8 μM) treatments.

Interestingly, when SH-SY5Y cells were simultaneously treated with Aβ42 and no cytotoxic LYC concentrations (8 and 1.6 μM) slight or no changes in cell viability and morphology were detected, strongly indicating a protective role of LYC against amyloid-β toxicity. The simultaneous exposure to Aβ and LYC concentrations ended up in a complete cell viability recovery (94% and 98%, for 8 µM and 1.6 μM, respectively) and a cell morphology preserving the neuronal processes typical of differentiated healthy SH-SY5Y cells (Figure 8). Compared to GAL, the effects exhibited by LYC were much more pronounced, since 2–3 order of magnitude higher GAL concentrations increase cell viability to a lower extent (77% for 500 µM) and do not restore the neuronal-like morphology altered by Aβ (Figure S4). 

## 4. Conclusions

In this study, we explored the chemical activity of LYC, a natural alkaloid well studied by the scientific community for its interesting pharmacological properties. To address this issue, we investigated (i) LYC interaction with Aβ by NMR and CD spectroscopy, (ii) LYC ability to prevent ROS production in Cu(II)/ascorbate systems and (iii) LYC protection against induced Aβ-toxicity in differentiated SH-SY5Y cell lines. The obtained data were also validated by the comparative analysis with GAL, a natural alkaloid well known as an active pharmaceutical ingredient (API) approved for the treatment of the early AD stages. By using an atomic and molecular approach we found out that both GAL and LYC are able to interact with the N-terminal region of Aβ through an electrostatic interaction between the positively charged N atom (GAL/LYC) and the Glu/Asp carboxylate groups of Aβ (Asp1, Glu3, Asp7 and Glu11). These interactions, further associated with Aβ structural rearrangements, encourage the continuation of the studies on LYC alone or in combination with other natural compounds [82] which are able to regulate several target proteins associated with the multifactorial AD etiology.

## 5. Materials and Methods

### 5.1. Materials

B-27™ Supplement (50X), serum free, Neurobasal™ Plus Medium and Glutamaxl were supplied by Thermo Fisher Scientific (Waltham, MA, USA). Galantamine hydrobromide from Lycoris sp (≥94% HPLC) and Lycorine hydrochloride (≥98% HPLC) were purchased from Sigma-Aldrich (Schnelldorf, Germany). Human Aβ42 and Aβ40 were supplied by GenScript Biotech (Rijswijk, Netherlands). All the other media and solvents used for cell culture and differentiation, mouse immortalized fibroblasts NIH3T3 and human neuroblastoma cell line SH-SY5Y were purchased from Sigma-Aldrich (Schnelldorf, Germany). Organo-tin stabilized polyvinyl chloride (PVC-org. Sn) was supplied by US Pharmacopeia (Rockville, MD, USA). 

### 5.2. Sample Preparation

Lyophilized Aβ40 and Aβ42 peptides were solubilized according to previous protocols by dissolving the peptides in NaOH 10 mM [73,83]. The samples were then diluted with phosphate buffer at pH 7.5 or EtOH/buffer mixture (60/40).

### 5.3. NMR Experiments 

NMR spectra were performed at 14.1 T with a Bruker Avance III Spectrometer (Bruker) operating at controlled temperature (± 0.2 K) and using a 5 mm BBI probe. All the experiments were collected at 298 K. Chemical shifts were referenced to external 3-(Trimethylsilyl)propionic-2,2,3,3-d_4_ acid sodium salt (TMSP-d_4_). TOCSY and NOESY spectra were recorded by using standard pulse sequences. NOESY spectra were acquired with a mixing time ranging from 100 to 300 ms in order to optimize the best value for NOE transfer. NMR spectra were processed and analyzed by using the TopSpin 3.6 software. Residual water signal was suppressed by excitation sculpting pulse program, applying a selective 2 ms long square pulse on water [84]. Stock GAL and LYC solutions (5 mM) in phosphate buffer 20 mM pH 7.5, were freshly prepared and used to obtain the final desired alkaloid concentration (0.25–0.15 mM) in phosphate buffer 20 mM pH 7.5 with 10% of D_2_O. Aβ40-containing samples were prepared by diluting peptide solutions (vide supra) with alkaloid-buffered solutions or EtOD/buffer mixture (60:40) to reach an Aβ40 concentration ranging from 0.075 to 0.180 mM in the NMR tube.

### 5.4. CD Studies

CD spectra were acquired on a Jasco J-815 spectropolarimeter at room temperature. A 1 cm cell path length was used for data between 190 and 260 nm, with a 1 nm sampling interval. Four scans were collected for every sample with scan speed of 100 nm min^−1^ and bandwidth of 1 nm. Baseline spectra were subtracted from each spectrum and data were smoothed with the Savitzky-Golay method [85]. Data were processed using Origin 5.0 spread sheet/graph package. The analyzed samples were prepared by diluting peptide solutions (vide supra) with phosphate-buffered solutions or EtOD/buffer mixture to have a final concentration: Aβ40/Aβ42 = 3.0 × 10^−6^ M. The GAL/LYC concentration 2.4 × 10^−6^ M in the CD cuvette was obtained by using stock GAL/LYC solutions.

### 5.5. UV-VIS Studies

The analyzed samples were prepared from GAL, LYC, ascorbate stock solution and diluted with phosphate buffer (3 × 10^−3^ M pH 7.4) or distilled water to have a final concentration of 5.0 × 10^−5^ M. Copper addition was performed by using stock solution of Cu(NO_3_)_2_ or CuSO_4_ in water. The absorption spectra and the kinetic curves (60 min, 3600 s) were recorded on a Perkin Elmer Lambda 900 UV/VIS/NIR spectrophotometer.

### 5.6. Differentiation of SH-SY5Y Cells

SH-SY5Y neuroblast-like cells were differentiated in neurons following the procedure reported by Shipley et al. [81]. Briefly, the maintenance culture at third passage was split when cells reached about 70–80% confluency using 2 mL of 1× 0.05% Trypsin-EDTA solution for three min in incubator. Then, trypsin was quenched by adding 10 mL of Basic Growth Medium (BGM). The content of the flask was transferred in a 15 mL conical tube and centrifuged at 1000 rcf for 2 min at room temperature. The supernatant was gently aspirated, and the pellet resuspended in 5 mL BGM. Cells were counted by using a hemocytometer and diluted with BGM to 50,000 cells/mL. Two mL of cell suspension were plated per each 35 mm^2^ Petri dish for a total of 100,000 cells per dish and placed into an incubator.

Differentiation continued according to the steps listed below.

- Day 1: BGM was removed, and Differentiation Media 1 (DM 1) was added to each Petri dish;
- Days 3 and 6: DM 1 was changed;
- Day 7: cells were split as described before, suspended in DM 1 and plated into new 35 mm^2^ Petri dishes;
- Day 8: DM 1 was removed and Differentiation Medium 2 (DM 2) was added at each dish;
- Day 10: cells were split, suspended in DM 2, seeded in 35 mm^2^ extracellular matrix (ECM) coated plates and returned to incubator;
- Day 13: DM 2 was removed and Differentiation Medium 3 (DM 3) added;
- Days 14 and 17: DM 3 was changed with fresh medium. At Day 18 neuronal cultures were ready for use.

### 5.7. Cytotoxicity Evaluation of Test Compounds towards Differentiated SH-SY5Y Cells

The cytotoxicity of different concentrations of Aβ (2 and 5 µM), LYC (16, 8, 1.6 µM) and GAL (500, 250, 50 µM) towards differentiated SH-SY5Y cells was evaluated in order to select the most suitable values to be tested with neuronal cells. The concentration of Aβ were chosen considering the data reported by Stroud et al. demonstrating that the amyloid peptide was toxic to HeLa and PC-12 neuronal cells at concentrations as low as 2.25 μg/mL [86]. Then, the cytotoxicity of LYC-Aβ and GAL-Aβ was studied. In particular, the following concentrations were analyzed: LYC 16 µM + Aβ 2 µM, LYC 8 µM + Aβ 2 µM, LYC 1.6 µM + Aβ 2 µM, GAL 500 µM + Aβ 2 µM, GAL 250 µM + Aβ 2 µM, GAL 50 µM + Aβ 2 µM. The stock solution of Aβ was prepared in Et-OH 60%, those of GAL and LYC in distilled water, and then properly diluted with DM 3 until the desired concentration to be tested with the cells was obtained. 

A total of 2 mL of each of the test solutions were then added to each Petri dish and incubated for 24 h at 37 °C and 5% CO_2_. At the end of the incubation, the viability of the differentiated SH-SY5Y cells was assessed by the NRU test (see infra). Each sample was tested in three replicates.

For the statistical analysis, multiple comparison was performed by one-way ANOVA and individual differences tested by Fisher’s test after the demonstration of significant intergroup differences by ANOVA. Differences with *p* < 0.05 were considered significant.

### 5.8. Evaluation of Cell Viability by Neutral Red Uptake (NRU) Test

At the end of the incubation, the routine culture medium was removed from each well, and cells were carefully rinsed with 1 mL of pre-warmed D-PBS. Multiwells were then gently blotted with paper towels. A total of 1.0 mL of NR Medium (1.0 mL NR Stock solution + 99.0 Routine Culture Medium pre-warmed to 37 °C) was added to each well and further incubated at 37 °C, 95% humidity, 5.0% CO_2_ for 3 h. The cells were checked during the incubation for NR crystal formation. At the end of incubation, the NR Medium was removed, cells were carefully rinsed with 1 mL of pre-warmed D-PBS. Then, the PBS was decanted and blotted from the wells and exactly 1 mL of NR Desorb solution (1% Glacial acetic acid solution + 50% + 49% H_2_O) was added to each sample. Multiwells were then put on a shaker for 20–45 min to extract NR from the cells. During this step the samples were covered in order to protect them from light. After 5 min from the plate shaker removal the absorbance was read at 540 nm by a UV/Visible spectrophotometer (Varian Cary 1E).

## Data Availability

Not applicable.

## Supplementary Materials

The following supporting information can be downloaded at: https://www.mdpi.com/article/10.3390/ijms24032500/s1.
