# Supplementary material for: A Comparative Study between Lycorine and Galantamine Abilities to Interact with AMYLOID β and Reduce In Vitro Neurotoxicity
Source: Int J Mol Sci. 2023 Jan 28;24(3):2500. doi: 10.3390/ijms24032500 (PMC9916559; doi:10.3390/ijms24032500)
Supplement: Supplementary file 1 [file ijms-24-02500-s001.zip › ijms-2102140-supplementary.pdf]

Supplementary Material

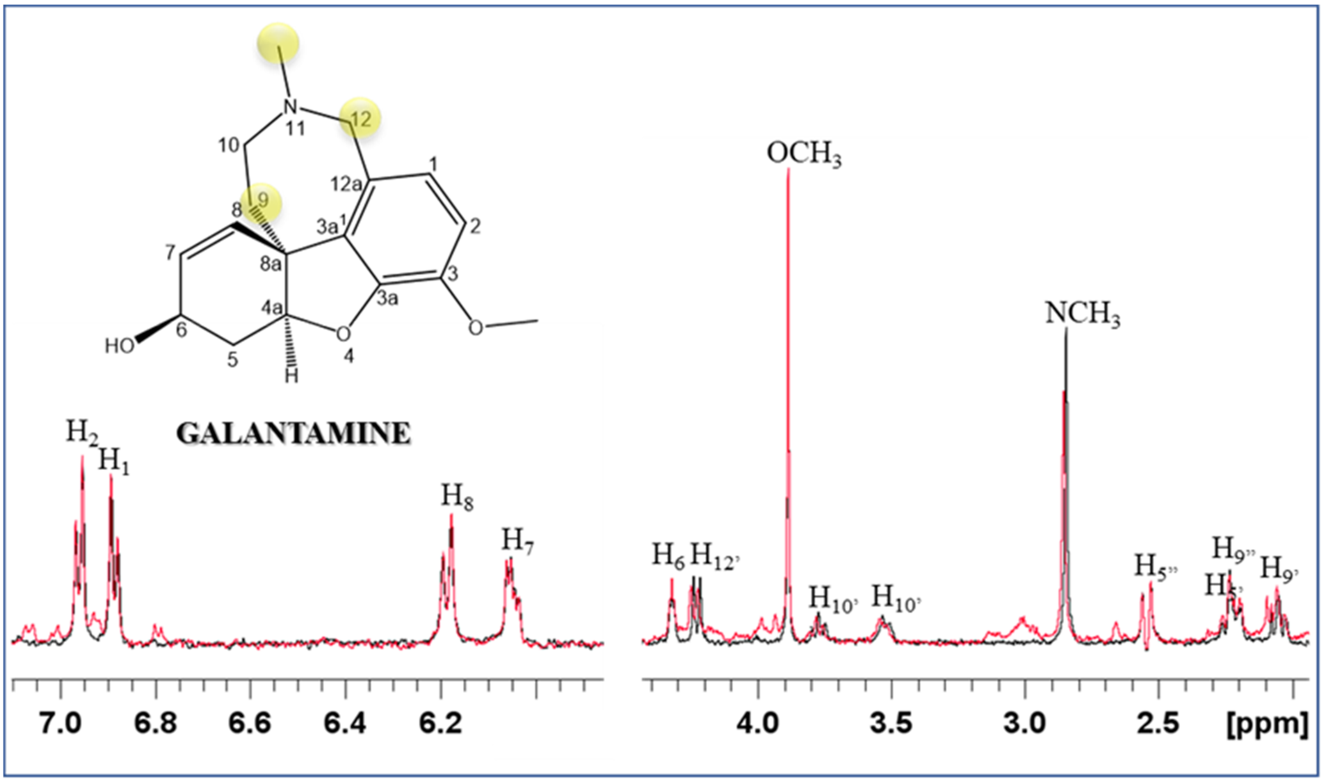

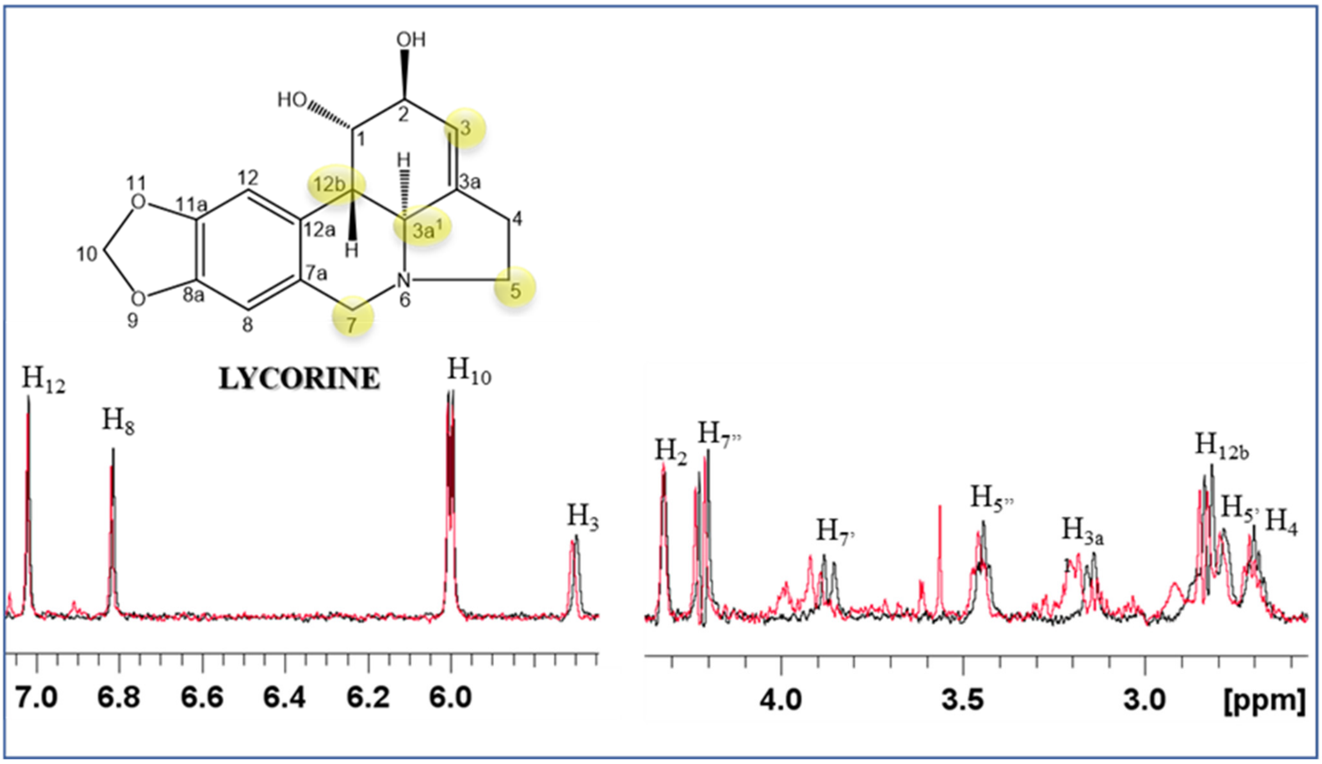

Figure S1. Selected regions of ^{1}H spectra of GAL (upper trace) and LYC (lower) in absence (black lines) and in presence of 0.25 eqs. of Aβ40 after 48h incubation (red lines). Alkaloid concentration 0.25 mM, phosphate buffer 20 mM, pH 7.5, T = 298K.

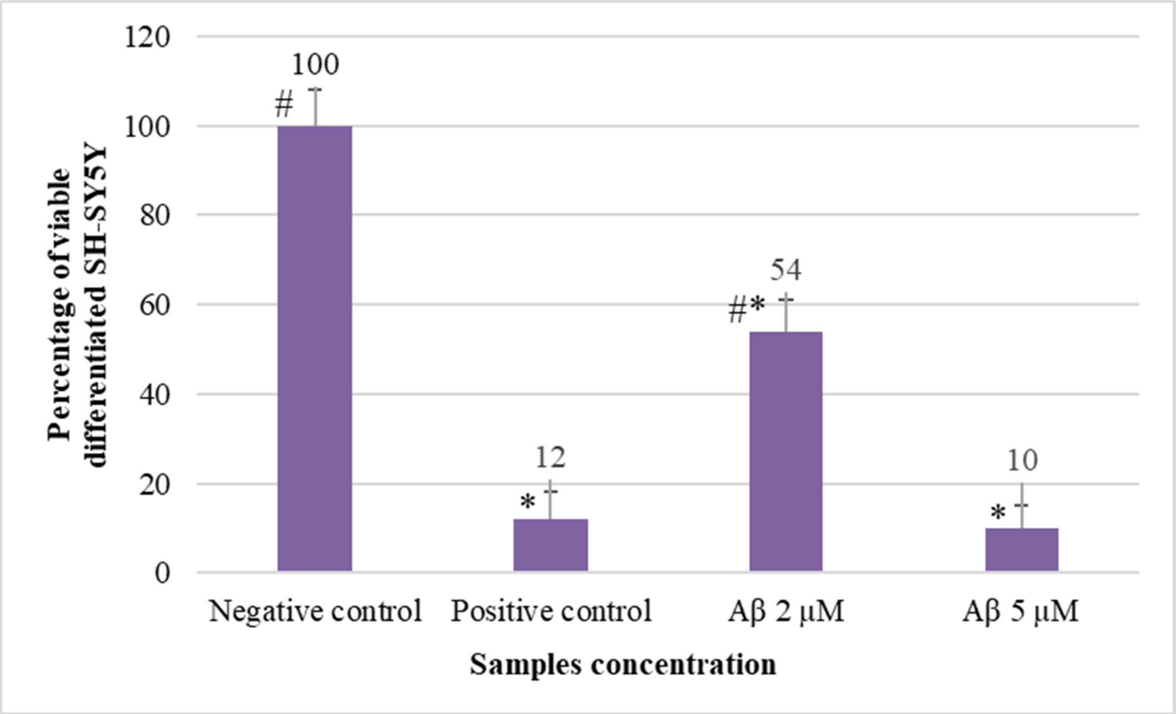

Figure S2. Percentage of viable differentiated SH-SY5Y as a function of Aβ concentration, as determined by the Neutral Red Uptake. Data are mean ± SD of three replicates for each sample. *Values are statistically different versus negative control (complete medium), $p < 0.05$. # Values are statistically different versus positive control (PVC-org.Sn), $p < 0.05$.

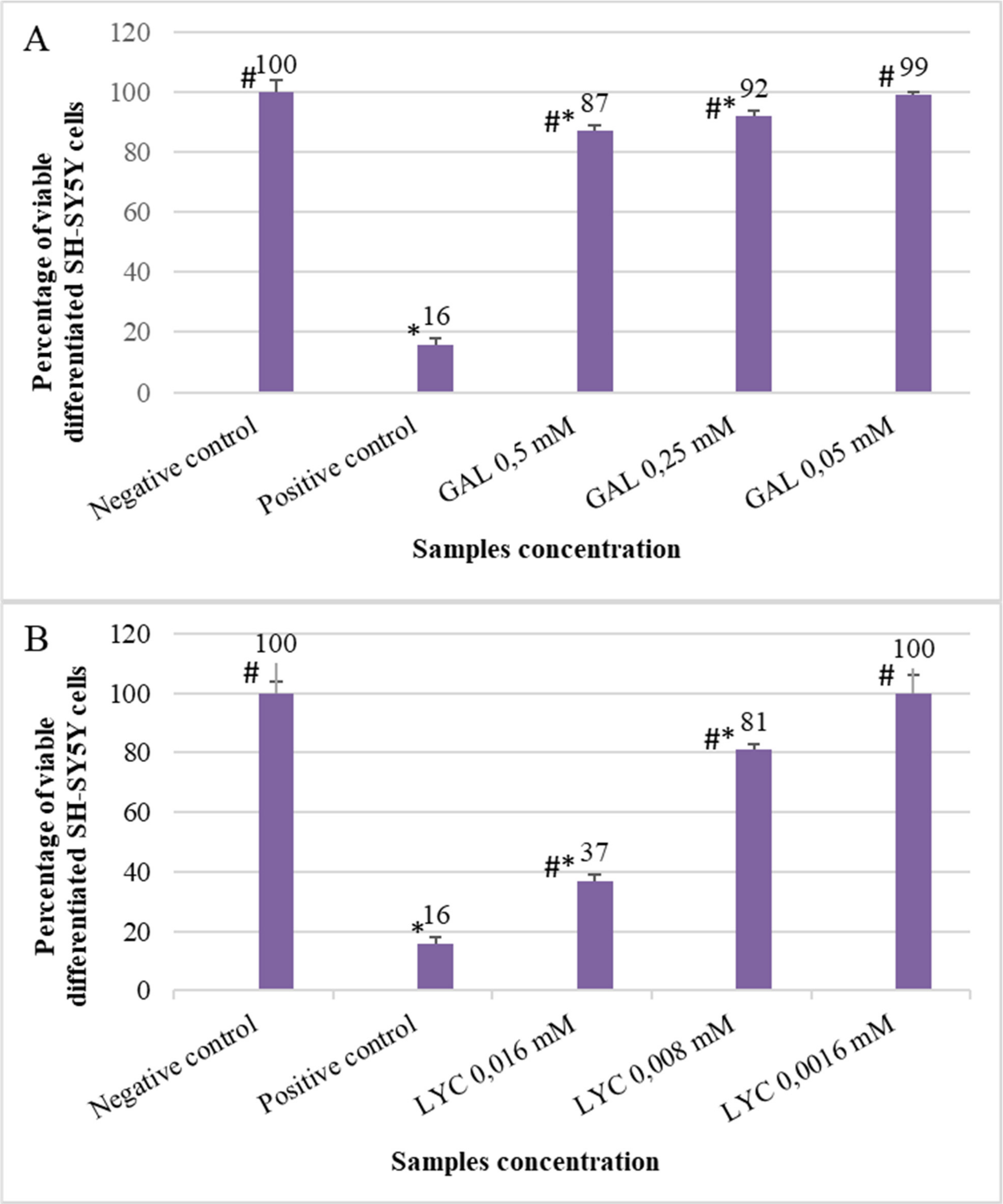

Figure S3. Percentage of viable differentiated SH-SY5Y as a function of (A) GAL and (B) LYC concentration, as determined by the Neutral Red Uptake. Data are mean ± SD of three replicates for each sample. *Values are statistically different versus negative control (complete medium), $p < 0.05$. # Values are statistically different versus positive control (PVC-org.Sn), $p < 0.05$.

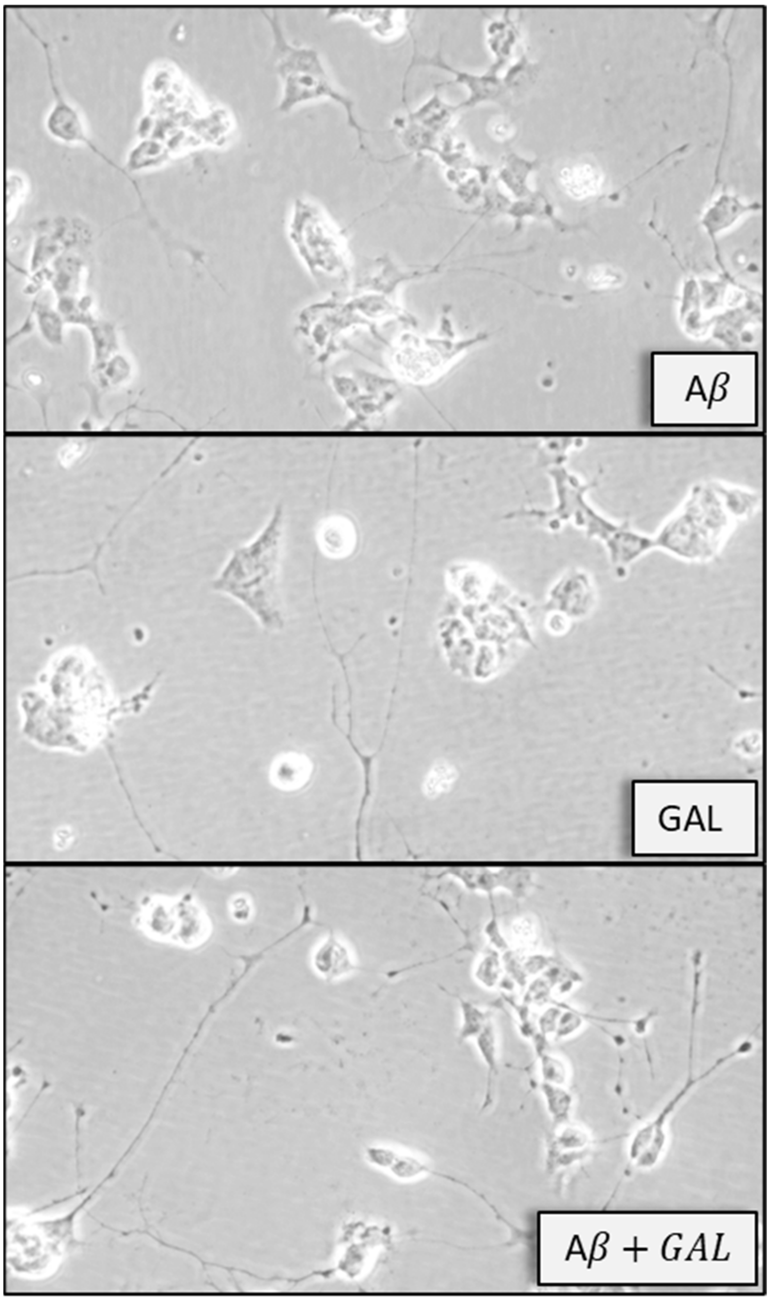

Figure S4. Optical microscope images of differentiate SH-SY5Y in contact with Aβ 2 µM; GAL. 500 µM; Aβ 2 µM + GAL 500 µM. Magnification 40x; scalebar 5 µm.
